# Posterior Colpotomy in Ectopic Pregnancy Management: A 12-Year Surgical Analysis

**DOI:** 10.7759/cureus.73366

**Published:** 2024-11-10

**Authors:** Angela Encarnação Sousa Silva, António Baptista Vilaça

**Affiliations:** 1 Gynecology and Obstetrics, Unidade Local de Saúde do Médio Ave, Vila Nova De Famalicão, PRT; 2 Gynecology and Obstetrics, Unidade Local de Saúde do Médio Ave, Vila Nova de Famalicão, PRT

**Keywords:** colpotomy, ectopic pregnancy, minimally invasive surgical procedures, tubal pregnancy, vaginal surgery

## Abstract

Introduction and objective: The management of ectopic pregnancies has evolved from traditional laparotomy to minimally invasive techniques, with posterior colpotomy emerging as a viable alternative. This study explores the safety and efficacy of posterior colpotomy in tubal ectopic pregnancy management.

Methods: A retrospective analysis of 16 cases employing posterior colpotomy for ectopic pregnancy treatment was conducted from 2009 to 2021.

Results: Posterior colpotomy demonstrated success in all 16 cases, with no intraoperative complications, short surgical times (15-65 minutes), and minimal blood loss. One patient (6.25%) experienced postoperative complications, necessitating transfusion. Most patients (87.5%) were discharged the following day, expressing high satisfaction.

Conclusion: The 12-year surgical analysis supports posterior colpotomy as a safe and effective technique for managing tubal ectopic pregnancies. Its benefits, including minimized trauma, short operating times, and favorable patient outcomes, affirm its contemporary relevance. The practical advantages further emphasize its viability in the evolving landscape of ectopic pregnancy management. Larger-scale research is recommended for comprehensive comparisons with laparoscopy and laparotomy.

## Introduction

The landscape of ectopic pregnancy management has undergone significant evolution, transitioning from traditional laparotomy approaches to the contemporary era of minimally invasive surgery [1-3]. Historically, laparotomy dominated the treatment of ectopic pregnancies, but an alternative method using vaginal colpotomy emerged as a viable option [3,4]. This approach brought forth numerous advantages, encompassing its diagnostic capabilities, facile access to and visualization of the pelvic adnexa, feasibility for definitive treatment, shorter operating times, and acceptable morbidity rates [1].

The advent of advanced laparoscopic techniques precipitated a paradigm shift, favoring exclusively laparoscopic management and causing colpotomy to lose favor [3,4]. Nevertheless, the pursuit of even less invasive approaches prompted a reevaluation of transvaginal surgery [1]. Posterior colpotomy, characterized by a horizontal incision in the posterior vaginal fornix, has resurged as a minimally invasive technique comparable to laparoscopy [5]. This method facilitates direct entry into the pelvic peritoneal cavity, offering advantages such as superior cosmetic outcomes, reduced surgical trauma, blood loss, neuroendocrine stress, inflammatory response, postoperative pain, and faster recovery times [1,2,5].

This study aimed to present the experience of our hospital in utilizing posterior colpotomy for the surgical treatment of tubal ectopic pregnancy. Against the backdrop of evolving surgical techniques, we delve into the safety and efficacy of culdotomy, shedding light on its contemporary role in the surgical armamentarium.

## Materials and methods

A retrospective chart review was conducted with the approval of the Ethics Committee of Unidade Local de Saúde do Médio Ave. The investigation focused on patients who underwent posterior colpotomy for the treatment of ectopic pregnancy between January 1, 2009, and December 31, 2021.

Inclusion criteria encompassed cases of posterior colpotomy performed for the treatment of ectopic pregnancies within the specified period. Cases lacking postoperative follow-up data were excluded. Ethical considerations were upheld by including only patients who had previously granted informed consent for the use of their medical records. Medical records were examined, including operative notes and both inpatient and outpatient clinic documentation. Adverse events occurring within 45 days postsurgery were identified.

Surgical technique

The procedure begins with positioning the patient in the lithotomy position, with thighs flexed at 45°- 60° upward at the hips. A weighted speculum is placed against the posterior vaginal wall and the anterior vesical wall is displaced using a Breisky valve, exposing the uterine cervix. The posterior border of the cervix is then clamped and traction is applied. An incision is created at the junction of the mucosa of the posterior vaginal wall and the uterine cervix using Metzenbaum scissors, which provide direct access to the cul-de-sac. Free blood within the cavity is drained, and the affected uterine tube is evaluated using Deaver retractors. If necessary, traction is applied with Foerster sponge forceps to facilitate salpingectomy. The salpingectomy is performed using a curved clamp, and subsequently, tubal ligation is done with an endoloop. The pelvic cavity is washed with saline solution, and the colpotomy is closed using a running locked technique with a delayed absorbable suture.

Data were analyzed using SPSS software version 26 (Armonk, NY: IBM Corp.). Means or medians were calculated for continuous variables, while frequencies or proportions were calculated for categorical variables.

## Results

A total of 16 cases of ectopic pregnancy treated through posterior colpotomy were identified. The demographic and surgical characteristics are summarized in Table 1. Surgical times ranged from 15 to 65 minutes (median: 32.5), and estimated blood loss varied from minimal to 250 mL.

**Table 1:** Demographic and surgical characteristics.

Variable	N (%)
Maternal age (mean±SD)	31.3 (4.1)
Nulliparas	8 (50%)
Successful culdotomy	16 (100%)
Surgical time (median)	32.5 minutes
Estimated blood loss (range: 0-250 mL)	≈50 mL

All procedures were completed successfully without intraoperative complications. One patient (6.25%) experienced postoperative complications in the form of anemia requiring transfusion. The majority of patients (14 out of 16, 87.5%) were discharged the following day. At discharge, none of the women reported pain, and all expressed a high level of satisfaction with the procedure. Follow-up assessments revealed that 10 patients (62.5%) subsequently became pregnant, and all completed normal pregnancies resulting in successful deliveries.

## Discussion

The management of ectopic pregnancies has witnessed a transformative journey over the years, evolving from traditional laparotomy to the widespread adoption of minimally invasive techniques [1,3,4]. In this retrospective analysis, we focus on the utilization of posterior colpotomy as a minimally invasive approach for the surgical treatment of tubal ectopic pregnancy. This study aimed to contribute insights into the safety, efficacy, and contemporary role of posterior colpotomy in the evolving landscape of ectopic pregnancy management.

The shift from laparotomy to minimally invasive approaches, particularly laparoscopy, has been a hallmark in the management of ectopic pregnancies [2,3]. However, the resurgence of posterior colpotomy as a viable alternative warrants attention [1]. This technique offers significant advantages, including superior cosmetic outcomes, as it allows the surgeon to remove the ectopic pregnancy without any visible incisions, providing a more satisfactory cosmetic result for the patient. Additionally, posterior colpotomy is associated with reduced surgical trauma, blood loss, neuroendocrine stress, inflammatory response, postoperative pain, and faster recovery times. These benefits align with the growing emphasis on patient-centered care and improved surgical experiences [4].

Our findings reveal that posterior colpotomy is a feasible and effective technique for the treatment of ectopic pregnancies. The successful completion of culdotomy in all cases, with minimal intraoperative complications and short surgical times, underscores the technical proficiency and safety of this approach. The absence of intraoperative complications aligns with the existing literature, emphasizing the safety profile of posterior colpotomy [3,4].

The postoperative period is critical in assessing the overall success and acceptability of any surgical procedure. In the present study, the majority of patients were discharged the day following surgery, reflecting the quick recovery associated with posterior colpotomy. Furthermore, the absence of reported pain and the high satisfaction levels among patients at discharge highlight the favorable postoperative outcomes associated with this technique.

While the overall complication rate in this study was low, it is essential to acknowledge and address the case of postoperative anemia requiring transfusion in one patient. This incident underscores the importance of continued vigilance in monitoring postoperative outcomes. Potential complications associated with the procedure, although infrequent, may include infection, hemorrhage, and injury to surrounding structures. In this particular case, the postoperative anemia was associated with a ruptured ectopic pregnancy, highlighting the complexities that can arise even in minimally invasive procedures.

Nonetheless, accessing the peritoneal cavity through the posterior route presents certain limitations. Specifically, the procedure should be avoided in individuals with cul-de-sac diseases, such as endometriosis or suspected adhesions. This underscores the significance of thorough clinical examinations before contemplating the use of this technique, particularly in the context of ectopic pregnancy management [6].

Our surgical analysis of posterior colpotomy in the management of ectopic pregnancy reinforces its status as a safe and effective minimally invasive technique. The low complication rates and quick recovery times contribute to its contemporary role in the surgical armamentarium for ectopic pregnancy management. As surgical techniques continue to evolve, posterior colpotomy remains a valuable option, offering benefits that align with the broader goals of patient-centered care and enhanced surgical experiences.

Nevertheless, we recognize the necessity for additional research involving larger sample sizes to enhance the robustness of these findings and establish a thorough comparison between posterior colpotomy, laparoscopy, and laparotomy in the management of ectopic pregnancy.

## Conclusions

In conclusion, our 12-year surgical analysis strongly endorses the viability of posterior colpotomy as a safe and effective alternative for managing tubal ectopic pregnancies. The method's notable benefits, including minimized surgical trauma, shorter operating times, and favorable patient outcomes, firmly establish its contemporary significance in the evolving landscape of ectopic pregnancy management. Additionally, posterior colpotomy can offer advantages over laparoscopy, such as lower costs associated with the use of inexpensive and simple surgical tools, as well as a reduced risk of complications related to port-site infections and surrounding organ injuries. The rapid rehabilitation, brief hospital stay, and more satisfactory cosmetic outcomes further underscore the practical advantages of this approach, making it a compelling option for clinicians and patients alike.
